# Cancer Neuroscience: From Local Neuro–Immune Microenvironments to Systemic Brain–Body Circuitry

**DOI:** 10.1002/mco2.71025

**Published:** 2026-09-11

**Authors:** Mingxuan Xie, Yu Zhao, Xinruo Xing, Yuqiang Liu, Jungang Zhao, Maojin Yao, Jun Tang, Jianzhu Zhao

**Affiliations:** ^1^ Department of Thoracic Surgery Sheng Jing Hospital China Medical University Shenyang China; ^2^ State Key Laboratory of Respiratory Disease, National Clinical Research Center For Respiratory Disease, National Center For Respiratory Medicine, Joint International Research Laboratory of Respiratory Health, Guangdong Basic Research Center of Excellence for Respiratory Medicine, Department of Thoracic Surgery and Oncology, Guangzhou Institute of Respiratory Health the First Affiliated Hospital of Guangzhou Medical University Guangzhou P. R. China; ^3^ China‐Portugal Artificial Intelligence and Public Health Technologies Joint Laboratory, Guangdong‐Hong Kong‐Macao Joint Laboratory of Respiratory Infectious Diseases, Guangdong Provincial Key Laboratory of Respiratory Disease Research Guangzhou Medical University Guangzhou China; ^4^ Department of Oncology Sheng Jing Hospital, China Medical University Shenyang China

**Keywords:** blood–brain barrier, nervous system, neuro‐oncological therapies, neuro–tumor interactions, tumor microenvironment

## Abstract

Classic oncological models have largely restricted cancer pathogenesis to autonomous genetic mutations within localized cells. Current research, however, reframes solid tumors as structurally integrated “pseudo‐organs” that rely on host neural networks for survival. This review examines the reciprocal signaling networks connecting nervous, immune, and malignant cells within the tumor microenvironment. At the tissue level, we outline how tumors construct de novo neural architecture via axonogenesis, cellular plasticity, and the assembly of functional neuro‐neoplastic synapses. Expanding beyond the local niche, we describe the systemic brain–body circuitry. We specifically address how central nervous system pathways respond to tumor‐derived cues and how chronic stress signaling disrupts physiological homeostasis. To explain the associated immune evasion, we analyze how sensory neuropeptides, particularly CGRP and, more broadly, Substance P–NK1R signaling, contribute to immune dysfunction, tumor‐promoting inflammation, and therapeutic resistance. Finally, we discuss the clinical implications of these pathways. Rather than relying on nonspecific structural denervation, future therapeutic strategies should focus on targeted “neural reprogramming” to uncouple tumors from their neural dependencies and restore antitumor immunity while minimizing neurotoxicity. Ultimately, this review conceptualizes solid tumors as systemically integrated entities, establishing a framework for precision neural reprogramming to overcome immune evasion and therapeutic resistance.

## Graphical abstract

This review outlines the cancer neuroscience paradigm, spanning systemic brain–body circuitry and local neuro–immune–tumor microenvironments. We discuss how tumors promote axonogenesis and pseudo‐synapse formation, and how sensory neuropeptides, such as CGRP, contribute to CD8+ T‐cell exhaustion. We further examine translational frontiers, with particular emphasis on spatial multi‐omics and “precision neural reprogramming” strategies, including β‐blockers combined with immunotherapies, to overcome neuronally mediated therapeutic resistance.

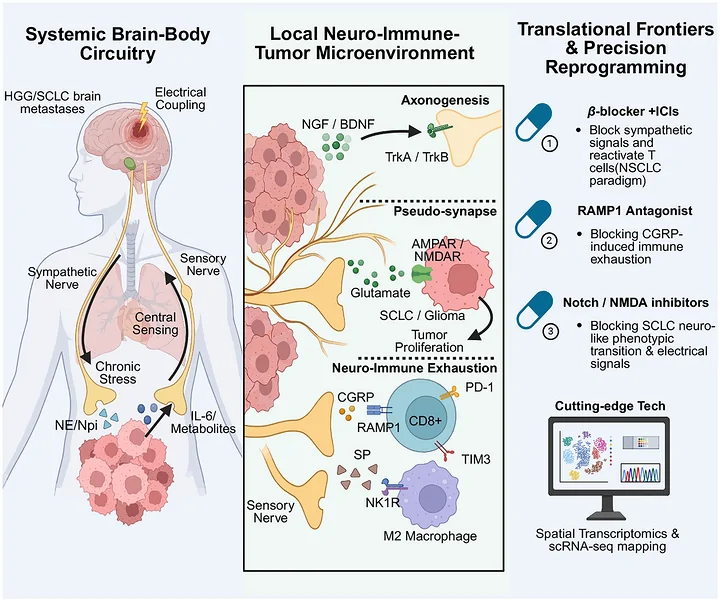

## 1 Introduction

Traditionally, cancer has been viewed primarily as a genetic disorder driven by cell‐autonomous mutations. However, this view is being reshaped by a paradigm shift that defines tumors as complex, innervated pathophysiological entities. Solid tumors are increasingly understood to actively recruit and integrate with the host nervous system [1, 2], suggesting that neuro‐neoplastic interactions may represent a distinct hallmark of cancer [3], comparable to angiogenesis [4]. This relationship is not simply passive [1, 2]. Experimental studies have shown that autonomic nerve fiber infiltration contributes to tumor initiation and progression in prostate cancer models [5], while basal cell carcinoma has been reported to arise preferentially within mechanosensory nerve‐rich niches [6].

Crosstalk between neural components and tumor cells is highly specific and bidirectional. Sympathetic signaling predominantly promotes tumor progression. In prostate cancer, adrenergic nerves induce an “angio‐metabolic switch” through β2‐adrenergic receptors, thereby altering endothelial metabolism and sustaining exponential tumor growth [7]. Furthermore, epinephrine‐mediated sympathetic activation promotes COX‐2‐dependent immunosuppression in myeloid cells, thereby shielding tumors from immune surveillance [8]. In contrast, parasympathetic signaling has complex and organ‐specific effects. For example, vagal denervation suppresses gastric tumorigenesis by inhibiting Wnt signaling [9], whereas cholinergic signaling through the α5‐nicotinic acetylcholine receptor drives metastasis in intrahepatic cholangiocarcinoma [10]. Moreover, activation of muscarinic M1 receptors can confer resistance to taxane‐based chemotherapy, directly linking neural signaling to treatment failure [11].

Sensory neurons add to the neural landscape of cancer. Sensory ablation can be used to delay the appearance of precursor lesions, suggesting a role for sensory neurons in early pancreatic ductal adenocarcinoma PDAC) carcinogenesis. In addition, recent research shows that surgical removal of sensory nerve fibers can help prevent mammary tumor metastasis, indicating that sensory fibers are involved in the process of metastasis [12]. Mechanistic studies are now underway to address the systemic and molecular mechanisms underlying these effects. Borniger [13] called cancer a “homeostatic challenge,” suggesting that sensory afferents sense cytokines and metabolites from cancer at the brain–body interface, and in that way disrupt systemic physiology. Wang et al. [14] went further in demonstrating how tumors can alter the immune microenvironment by leveraging homeostatic neural circuits, and the brain–body axis is emerging as a therapeutic frontier. Tumors remodel their local environment, rather than passively waiting for innervation: for instance, tumor‐derived exosomes can induce de novo innervation, or axonogenesis, which can provide a structural basis for high‐bandwidth neuro–tumor communication [15].

Cancer can be thought of as a systemic disease which affects the brain–body interface, beyond the local tumor microenvironment. It may be that chronic psychological stress is converted into physiological signals which remodel lymphatic vessels and promote tumor cell dissemination [16]. It could also promote metastasis by promoting the remodeling of the tumor microenvironment via neutrophils [17]. Also recently, direct neural pathways have been identified between the brain and the site of the tumor, indicating that central neural activity can impact peripheral tumor activity from afar [18]. Meanwhile, systemic treatments like methotrexate can have long‐lasting effects on glial regulation and thus can lead to pathological alterations in cognition, what is also called “chemobrain,” as well as further reshaping of the neural landscape [19].

In this review, we synthesize the existing evidence into a multiscale theoretical framework that traces the interactions between cancer and the nervous system, ranging from local neural remodeling within the tumor microenvironment to systemic brain–body neural circuits. Section 2 explores how tumors establish and reprogram local neural structures through axonogenesis, neurogenesis, glial plasticity, and synaptic or electrochemical integration. Section 3 extends this framework to long‐distance regulation. In this section, we focus on how tumor‐derived signals, chronic stress, and autonomic and neuroendocrine circuits disrupt homeostasis and reshape the tumor macroenvironment. In Section 4, we explore the neuro‐immune‐tumor interface. This section highlights the bidirectional interactions among the neural, immune, and tumor compartments, as well as their impact on immune evasion and tumor progression. Rather than treating molecular mediators—such as neurotransmitters, neuropeptides, and neurotrophic factors—as isolated topics, we weave them throughout the review. They serve as a central mechanistic thread, closely linking local neural remodeling, systemic regulation, and neuroimmune modulation. Finally, Section 5 translates this theoretical framework into clinical opportunities and challenges. We highlight the paradigm shift in therapeutic strategies from nonspecific neural denervation to precision targeting of tumor‐specific neural dependencies. This includes pathway‐targeted interventions, immunotherapy combinations, disease‐specific strategies, and clinical translation guided by biomarkers and safety considerations.

## 2 Neural Remodeling in the TME

**FIGURE 1:**
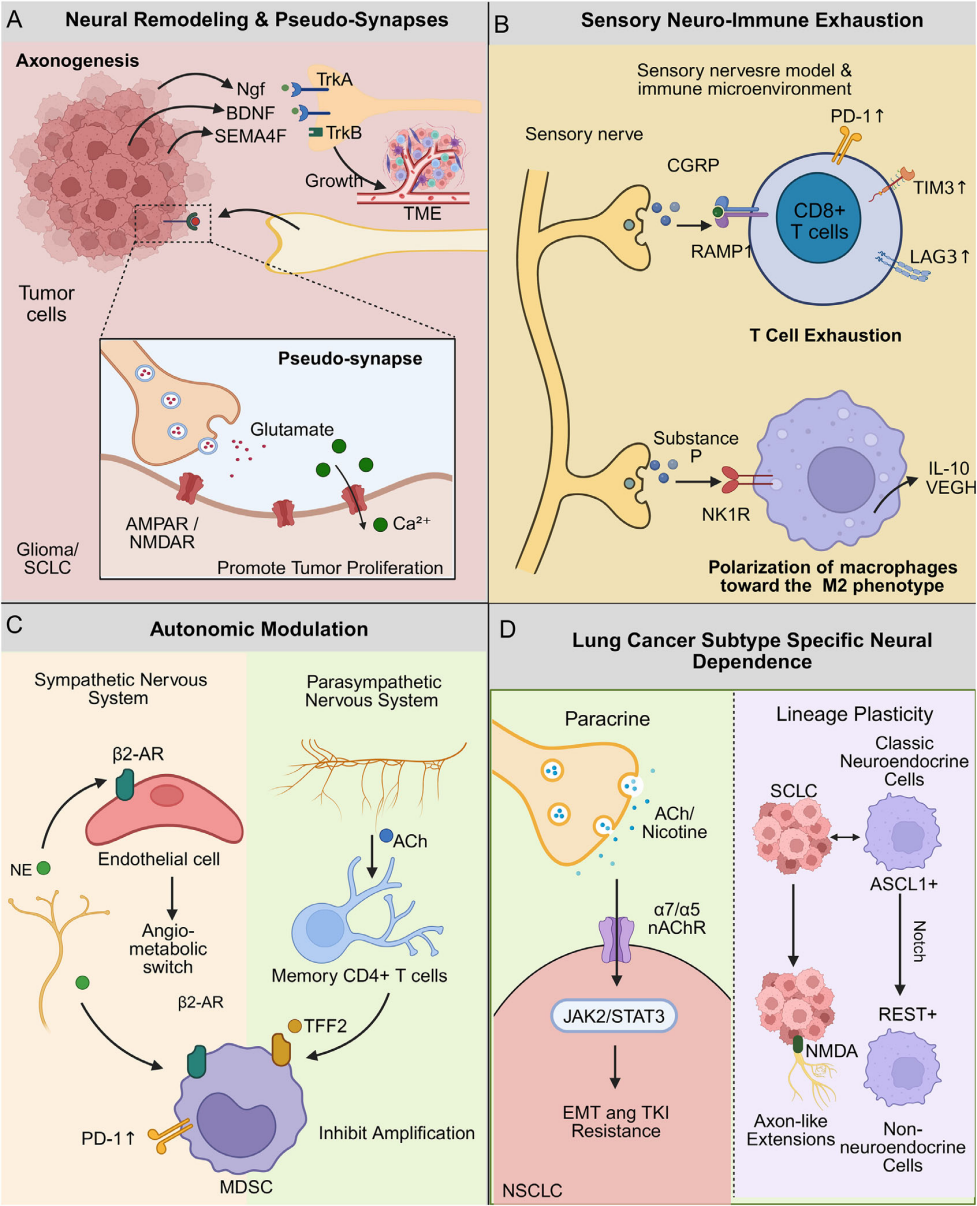
Molecular mechanisms of the local neuro–immune–tumor tripartite microenvironment. (A) Tumors actively recruit nerve fibers via neurotrophic factors (NGF, BDNF) and axonal guidance cues (SEMA4F) to drive axonogenesis, eventually forming glutamatergic pseudo‐synapses through AMPAR/NMDAR to promote direct proliferation. (B) Sensory nerves establish an immunosuppressive niche by releasing CGRP, which binds to RAMP1 on CD8+ T cells to drive profound exhaustion, and substance P (SP), which skews macrophages toward an M2‐like phenotype via NK1R. (C) Sympathetic nerves release norepinephrine (NE) to activate β2‐ARs on endothelial cells (triggering an angio‐metabolic switch) and MDSCs (enhancing immunosuppression), whereas vagal signaling can restrict MDSC expansion via TFF2. (D) Distinct neural dependencies in lung cancer subtypes: NSCLC predominantly relies on paracrine cholinergic signaling (α7/α5 nAChR) to drive EMT and therapeutic resistance, whereas SCLC exploits Notch‐mediated lineage plasticity (ASCL1+ to REST+ transition) and forms intrinsic autocrine pseudo‐synaptic networks. NGF, nerve growth factor; BDNF, brain‐derived neurotrophic factor; SEMA4F, semaphorin 4F; CGRP, calcitonin gene‐related peptide; SP, substance P; NE, norepinephrine; MDSC, Myeloid‐derived suppressor cell; EMT, epithelial–mesenchymal transition.

Innervation of the tumor microenvironment (TME) is not a random or passive event, but a coordinated biological process [5]. Increasing evidence suggests that solid tumors, especially those arising in densely innervated organs such as the lung, can actively generate de novo neural architecture through a process referred to as neoneurogenesis [20]. This structural remodeling provides the physical substrate for bidirectional neuro‐oncological communication and involves four progressively complex mechanisms: axonogenesis, central neurogenesis, phenotypic reprogramming, and functional synaptic integration [20, 21, 22] (Figure 1).

### 2.1 Tumor Innervation: Axonogenesis and Neurogenesis

#### 2.1.1 Axonogenesis: The Angiogenic Analogy and Chemotactic Signaling

##### 2.1.1.1 Neurotrophin Signaling

The best‐characterized mediator of tumor‐associated axonogenesis is nerve growth factor (NGF) [22]. In breast and prostate carcinomas, tumor cells overexpress NGF, which binds to high‐affinity TrkA and p75NTR receptors on extending sympathetic and sensory nerve terminals, thereby stimulating actin polymerization within neuronal growth cones and promoting neural recruitment [7]. Concurrently, NGF acts on tumor cells in an autocrine or paracrine manner, promoting their survival through PI3K/AKT signaling [23]. Importantly, recent precision oncology studies have shown that proNGF, the precursor form of NGF, correlates more strongly with high Gleason scores than mature NGF and promotes tumor invasion through p75NTR signaling [24]. Additionally, brain‐derived neurotrophic factor (BDNF) and glial cell line‐derived neurotrophic factor (GDNF) function as critical co‐regulators of tumor innervation. Although NGF signaling has been reported across multiple tumor types, lung adenocarcinoma (LUAD) appears to show distinct neural dependencies. In PDAC, tumor‐derived BDNF acts on TrkB‐expressing sensory neurons and increases tumor innervation density [25].

##### 2.1.1.2 Hijacking Guidance Cues

Embryonic axon guidance molecules are misexpressed by tumors. In the prostate TME, Semaphorin 4F (SEMA4F) is a repulsive guidance cue, but has been repurposed as a neuroattractant for sympathetic nerve fibers [26]. Also, Netrin‐1, a protein that is related to laminins, is overexpressed in colorectal and pancreatic cancers. The binding of Netrin‐1 to DCC or UNC5B receptors on enteric neurons promotes entry of these neurons into the tumor core and provides anti‐apoptotic support to these neurons, maintaining a pro‐tumorigenic neural niche [27, 28].

In addition to the SEMA4F and Netrin‐1 axes, recent progress in the field of neuro‐oncology has led to the discovery of a larger repertoire of axon guidance molecules that are also subverted by malignant cells. The Slit/Robo signaling pathway and the Ephrin/Eph receptor family have been recently demonstrated to play important roles in the coordination of tumor innervation in a variety of tumors, such as gastrointestinal and thoracic tumors [29, 30, 31, 32, 33, 34, 35]. In addition, recent studies have identified a growing list of neurotrophic molecules such as Artemin, glial cell line‐derived neurotrophic factor (GDNF), and neurotrophin‐3 (NT‐3) which act as strong chemoattractants, and which cooperate to create the perineural niche [36, 37, 38, 39, 40] (Figure 1).

#### 2.1.2 Neurogenesis: Systemic Recruitment of Central Progenitors

Lineage‐tracing studies challenge the classical view that tumor innervation arises solely from local axonal sprouting [41] and reveal a systemic mechanism in which neural progenitor cells (NPCs) are recruited from the central nervous system (CNS) [42].

##### 2.1.2.1 The Brain–Tumor Axis

Mauffrey et al. [42] showed that in prostate cancer, doublecortin‐positive (DCX+) neural progenitor cells (NPCs) from the brain's subventricular zone (SVZ) enter the circulation. These NPCs cross the blood–brain barrier (BBB), circulate through the bloodstream, and infiltrate both primary tumors and metastatic sites [43].

##### 2.1.2.2 Differentiation and Integration

After extravasation, these CNS‐derived neural progenitor cells (NPCs) differentiate into functional adrenergic neurons [42]. These results indicate that tumors can remotely utilize neurogenic niches in the brain to form their own neural networks, effectively bypassing the rate‐limiting constraints imposed by local axonal growth [20, 42].

### 2.2 Neural Reprogramming and Glial Modulation

#### 2.2.1 p53‐Mediated Transdifferentiation

Amit et al. [44] showed that exosomes released by the p53‐deficient tumor cells have a low miR‐34a content in head and neck squamous cell carcinoma (HNSCC). This deficiency causes transdifferentiation of the sensory neurons into an adrenergic phenotype, known as the “sensory‐to‐sympathetic switch, ” and allows these neurons to become capable of releasing norepinephrine and stimulating tumor growth [44, 45].

#### 2.2.2 Cholinergic Remodeling and Context‐Dependent Networks

Mechanisms of tumor innervation and neurotransmitter signaling are highly context‐dependent [46]. Acetylcholine (ACh), a classical neurotransmitter of the parasympathetic nervous system, can be utilized in autocrine loops by cancer cells to drive progression [47]. In colon and stomach cancer, ACh binds to the muscarinic receptor CHRM3 (M3R), which transactivates the epidermal growth factor receptor (EGFR) [48, 49, 50]. This transactivation leads to the activation of the MAPK/ERK and PI3K/AKT pathways in tumor cells and stimulates the production of matrix metalloproteinases (such as MMP7) that break down the extracellular matrix, allowing tumor cells to invade [51, 52, 53].

Mechanisms of tumor innervation in gastric cancer are quite different [9, 54]. Hayakawa et al. [54] showed that cholinergic (vagal) nerves are essential in the development of gastric tumors. Gastric stem cells secrete NGF, which is necessary to recruit vagal nerve fibers. Acetylcholine released from these fibers, in turn, stimulates proliferation of the epithelia via M3 muscarinic receptors, creating a feed‐forward niche that promotes stem cell expansion [54, 55].

However, the biological effects of neurotransmitters seldom remain consistent from one context to another [46]. In pancreatic ductal adenocarcinoma (PDAC), cholinergic signaling exerts an opposing effect through the activation of the M1 receptor (CHRM1) [56]. In this situation, ACh signaling paradoxically inhibits the MAPK/EGFR pathway, which reduces the CD11b+ myeloid cell niche and culminates in the suppression of liver metastasis [56].

Lung cancer exhibits yet another distinct cholinergic dependency. In non–small cell lung cancer (NSCLC), ACh and nicotine have a strong affinity for the α7 and α5 nicotinic receptor subunits [57, 58]. This binding induces epithelial‐mesenchymal transition (EMT) and reprograms signaling pathways—such as JAK2/STAT3 and Ras/Raf/MEK/ERK—allowing the tumor to evade EGFR‐targeted therapies [10, 57, 59].

#### 2.2.3 Neuroendocrine Mimicry and Lineage Plasticity

Neuroendocrine tumors (NETs) are prototypical examples of “neural mimicry”, and it is instructive to examine them to fully understand the concept [60]. In contrast to traditional solid tumors, which have to recruit peripheral nerves, classic NETs (gastroenteropancreatic NETs) are already provided with neuronal machinery. These tumors are able to synthesize and secrete biogenic amines, primarily serotonin (5‐hydroxytryptamine, 5‐HT), in large quantities [61]. In the TME, 5‐HT is not only a byproduct that is responsible for systemic carcinoid syndrome, but also a key factor that regulates the microenvironment. Serotonin stimulates strong desmoplasia (fibrous stroma) and neoangiogenesis, creating a protective microenvironment for the tumor via activation of 5‐HT receptors on stromal fibroblasts and endothelial cells [62].

Furthermore, the neuroendocrine phenotype is not simply a pathological–histological entity, but in fact an adaptive evolutionary strategy of nonneural tumors. One example is in the case of prostate cancer. A fraction of adenocarcinoma cells undergo large phenotypic reprogramming under strong selective pressure from androgen deprivation therapy (ADT) to give rise to a treatment‐induced neuroendocrine prostate cancer (t‐NEPC) [63, 64]. It is in this process that these cells completely become independent of the androgen receptor [63]. Rather, they acquire a “neuronal‐like” phenotype and secrete potent neuropeptides, including bombesin and neurotensin. These peptides act through robust autocrine and paracrine loops that sustain their androgen‐independent growth, as well as the survival of neighboring therapy‐sensitive cells, thereby contributing to therapeutic resistance [65, 66].

#### 2.2.4 Schwann Cell Plasticity

Tumor‐associated Schwann cells (TASCs) are characterized by marked phenotypic plasticity [67]. In pancreatic cancer, Schwann cells are organized into “tumor tracks,” which facilitate perineural invasion (PNI) [68, 69, 70].

### 2.3 Synaptic Integration and Electrochemical Communication

Beyond paracrine signaling, an important emerging concept in cancer neuroscience is the formation of direct electrochemical synapses between neurons and cancer cells [71, 72, 73] (Table 1).

**TABLE 1:** Summary of key molecular mediators and signaling pathways in the neural–tumor axis.

Mediator class	Specific molecule	Primary receptor	Downstream signaling pathways	Key biological effects	Representative cancer types	References
Neurotransmitter	ACh	CHRM3 (M3R)	EGFR transactivation, MAPK/ERK, PI3K/AKT	Promotes cell proliferation, MMP secretion, and invasion	Colorectal, gastric, NSCLC	[50, 52, 74]
Neurotransmitter	ACh	CHRM1 (M1R)	Inhibition of MAPK/EGFR	Suppresses CSC niche and myeloid infiltration	Pancreatic ductal adenocarcinoma	[56]
Neurotransmitter	Nicotine/ACh	α7, α5‐nAChR	JAK2/STAT3, CaMKII/GSK‐3β	Induces EMT, confers targeted therapy resistance	NSCLC, cholangiocarcinoma	[10, 75, 76, 77]
Neurotransmitter	NE	ADRβ2 (β2‐AR)	cAMP/PKA, suppression of COA6	Induces angio‐metabolic switch (aerobic glycolysis)	Prostate, Breast cancer	[7, 78, 79, 80, 81]
Neurotransmitter	Glutamate	NMDAR, AMPAR	Calcium influx, CaMKII/MAPK	Drives excitatory pseudo‐synapses, promotes brain metastasis	Glioma, SCLC, breast cancer	[71, 72, 73, 82]
Biogenic amine	Serotonin (5‐HT)	5‐HT2B Receptor	ERK/MAPK, TGF‐β signaling	Drives intense desmoplasia (fibrosis) and angiogenesis	Gastroenteropancreatic NETs	[62, 83]
Neuropeptide	SP)	NK‐1R	NF‐κB, MAPK, MMP‐2 upregulation	Promotes neurogenic inflammation and cellular motility	Pancreatic, breast cancer	[31, 84, 85, 86]
Neuropeptide	CGRP	CALCRL‐RAMP1	cAMP/PKA	Induces CD8+ T cell exhaustion, impedes tertiary lymphoid structures	Melanoma, squamous cell carcinoma	[87, 88, 89]
Neuropeptide	Bombesin/GRP	GRPR (BB2)	Ca^2^^+^ mobilization, PLC/PKC, MAPK, FAK/Src/Etk‐mediated AR activation	Promotes paracrine neuroendocrine signaling and androgen‐independent growth	Prostate cancer, especially CRPC/neuroendocrine‐associated prostate cancer	[83, 90, 91, 92, 93]
Neurotrophin	NGF	TrkA, p75NTR	PI3K/AKT, Hippo	Drives axonogenesis, establishes tumor‐nerve feedback loop	Gastric, prostate cancer	[5, 24, 54]
Neurotrophin	BDNF	TrkB	ERK/MAPK, PI3K/AKT	Suppresses anoikis, promotes EMT and distant metastasis	Breast, ovarian cancer	[94, 95, 96, 97]

#### 2.3.1 Neuro‐Neoplastic Synapses and Glutamatergic Pseudo‐Synapses

High‐grade gliomas(HGG) and brain metastases from breast cancers have been the subjects of pioneering studies which showed that cancer cells can trigger the development of functional synapse‐like interactions with neurons [71, 72, 98]. In HGG, glutamatergic neurons have been shown to form true synapses with glioma cells, which are dependent on AMPA receptors for their formation [99, 100, 101], and in breast cancer brain metastases, it has been found that NMDAR signaling is promoted near synapses with the brain metastatic cells, aiding synergistically with other mechanisms for the promotion of brain metastasis [72, 73] (Figure 1).

Importantly, functional synaptic interactions mediated by neurotransmitters are not confined to primary brain cancers [71, 73]. In extracerebral tumors, such as pancreatic cancer, sensory nerve endings form localized pseudo‐synapses with tumor cells. In this context, close synaptic proximity and localized glutamate release are required for tumor growth and perineural invasion, whereas diffuse neurotransmitter exposure alone is insufficient [102, 103].

#### 2.3.2 Electrical Coupling and Autocrine Loops in SCLC

These synapse‐like contacts are used for direct electrical transmission and can trigger calcium transients in cancer cells, leading to their invasion and metastatic colonization [71, 72, 73]. This mechanism has been most well characterized in brain tumors [71, 72] but may take place in peripheral carcinomas as well, pointing to a more complex neural integration in malignant tissues [102, 104] and the presence of pseudo‐synaptic interactions [102], involving voltage‐gated sodium channels (VGSCs) [105, 106] in these tissues.

Small‐cell lung cancer (SCLC) demonstrates extreme neuroendocrine lineage plasticity; it not only reacts to neural signals but actively mimics them [107, 108]. SCLC cells express and secrete neuropeptides intrinsically, resulting in strong autocrine loops [109]. Furthermore, SCLC cells integrate into central neural circuits, forming pseudo‐synapses with glutamatergic and GABAergic cortical neurons [110, 111]. In SCLC, neuronal activity activates NMDA and AMPA receptors, leading to widespread synaptic scaling, tumor‐cell depolarization, calcium influx, CaMKII activation, and Myc‐dependent proliferation and brain colonization [108, 110]. Aberrant electrical communication in these neuroendocrine tumors suggests the tumor acts as a “malfunctioning neural circuit,” a vulnerability that may be therapeutically disrupted using NMDA receptor antagonists [5, 82, 112, 113].

## 3 Systemic Neural Circuitry in Cancer

In addition to the local microenvironment, cancer is increasingly recognized as a systemic disease that actively perturbs organismal homeostasis through long‐range neural circuits [14, 114]. This “brain–tumor–body axis” enables bidirectional communication: peripheral tumors transmit molecular and sensory signals to the central nervous system (CNS), which in turn recalibrates neuroendocrine and autonomic outputs to promote tumor progression [14, 115]. In this section, we review how tumors exploit these long‐range circuits, from chronic stress pathways to direct synaptic integration, to support their survival [71, 72, 81, 116].

### 3.1 The Brain–Body Interface and Hijacking Homeostasis

The brain–tumor relationship is not just a passive response to disease, but an active perturbation of physiological homeostasis. New theories of the tumor consider the tumor as an “aberrant organ” [117, 118] taking advantage of the brain–body interface to satisfy metabolic requirements [119, 120, 121, 122].

#### 3.1.1 Central Sensing Mechanisms

**FIGURE 2:**
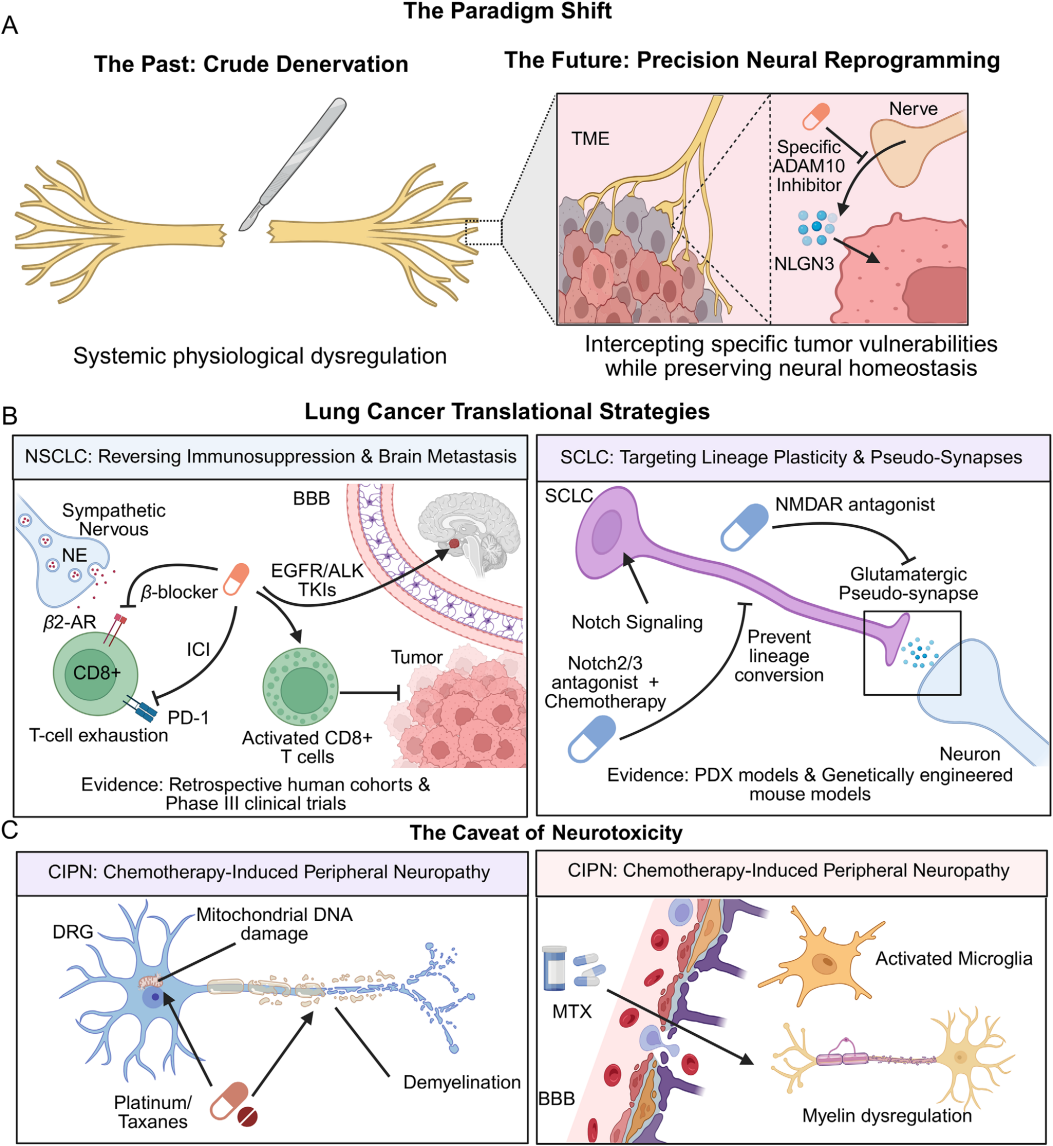
Systemic brain–body circuitry. **Incoming signals and central sensing**: Peripheral solid tumors actively release cytokines such as interleukin‐6 (IL‐6) and metabolic byproducts into the bloodstream. These signaling molecules travel upward and cross the blood–brain barrier (BBB), or act directly on periventricular organs (CVOs) with a leaky blood–brain barrier, enabling the central nervous system to detect peripheral tumor signals. **Central reprogramming**: The brainstem and hypothalamus undergo functional reprogramming upon receiving tumor signals. Neurons in the lateral hypothalamic area (LHA) and paraventricular nucleus (PVN)—which were originally responsible for energy regulation—are “repurposed,” thereby establishing, at the systemic level, a microenvironment that allows the tumor to evolve. **Signal transduction network**: The brain triggers the release of glucocorticoids via the hypothalamic‐pituitary‐adrenal (HPA) axis, mediating chronic stress and leading to systemic immunosuppression. At the same time, fibers from the sympathetic nervous system (SNS) physically infiltrate the tumor microenvironment (TME) and release norepinephrine (NE) locally. **TME remodeling and immune–metabolic transition**: Norepinephrine (NE) binds to β2‐adrenergic receptors (β2‐ARs) on endothelial cells, triggering a “vascular‐metabolic switch” that drives the endothelial metabolism toward aerobic glycolysis and promotes angiogenesis. Under sympathetic stress signals, tumor‐associated macrophages (TAMs) polarize into an M2‐like pro‐angiogenic state. Polarized TAMs secrete large amounts of VEGF and IL‐6, further driving tumor progression and shielding the tumor from immune surveillance.

One of the new questions in cancer neuroscience is how the CNS recognizes that there is a tumor [115, 123, 124]. Signals from the tumor, including cytokines like IL‐6 and other metabolites, can move across the BBB or directly act on the circumventricular organs (CVOs), which have a loose BBB [115, 125]. This allows the tumor to send out signals of its condition to the brainstem and hypothalamus [13, 125]. Upon receiving these signals, a particular set of neurons in the lateral hypothalamic area (LHA) and paraventricular nucleus (PVN) of the hypothalamus, which are normally involved with sleep and energy control [126], are repurposed. The final result is a recalibration of the system: the brain regulates whole‐body metabolism and inhibits antitumor immune responses via a permissive niche for tumor evolution [127, 128, 129] (Figure 2).

### 3.2 The Stress Axis: HPA and Sympathetic Drive

Psychological stress is a powerful systemic facilitator of cancer, mainly by stimulating the hypothalamic–pituitary–adrenal (HPA) axis and the sympathetic nervous system (SNS) [130, 131]. This cascade of release of glucocorticoids and catecholamines not only induces a “fight‐or‐flight” reaction but also actively changes the TME [131] (Figure 2).

The sympathetic nervous system actively reshapes the tumor microenvironment, primarily through the release of catecholamines such as norepinephrine (NE) [132, 133, 134]. In prostate cancer, NE binding to β2‐adrenergic receptors on endothelial cells induces a pronounced “angio‐metabolic switch,” whereby COA6 suppression inhibits oxidative phosphorylation and redirects endothelial metabolism toward aerobic glycolysis [7]. This metabolic shift meets the bioenergetic and biosynthetic demands of angiogenic sprouting. Under chronic stress, adrenergic signaling further enhances angiogenesis by promoting the release of VEGF and IL‐6 from tumor‐associated macrophages (TAMs) [79] (Figure 2).

Indeed, this systemic sympathetic activation targeting the endothelial cell β2‐adrenergic receptor triggers a metabolic switch from oxidative phosphorylation to glycolysis, which will directly lead to angiogenesis [7], as was seen in prostate cancer [135].

This is also a sympathetic signaling pathway that is crucial in metastasis [81]. Chronic stress has been known to lead to about 30 times more lung metastasis in breast cancer models [81]. This is because stress signals induce a “soil preparation” by attracting macrophages to the lungs, which makes them more receptive to the seeding of tumors later [136, 137]. In addition, this hormonal stress response creates an immunosuppressive environment. Stress hormones promote the elevation of the levels of these hormones, which in turn dampens type I interferon responses [138] and impairs the function of cytotoxic T‐cells [138, 139], which can make tumors resistant to immunotherapy—a major problem in the treatment of NSCLC [139, 140, 141, 142].

### 3.3 Central Regulation of Tumor Macroenvironment

CNS regulation of tumorigenesis occurs via two distinct mechanisms, depending on tumor location: remote modulation for peripheral cancers and direct integration for brain tumors.

#### 3.3.1 Remote Modulation of Peripheral Tumors

Certain neural reflexes have been shown to be critical regulators of immune modulation induced by a tumor [32, 115]. Brain innervation and activation of NPY2R/TRPV1‐enriched vagal sensory neurons to transmit afferent signals to brainstem nuclei and augment sympathetic efferent activity within the TME has been observed in models of lung adenocarcinoma [115]. This sensory‐to‐sympathetic pathway inhibits antitumor immunity by transmitting a β‐adrenergic receptor signal to alveolar macrophages, which has a negative effect on the response of tumor‐reactive T‐cells [115]. In all cases, genetic, pharmacological, or chemogenetic blockade of this circuit results in decreased lung tumor progression, likely by improving anticancer immune responses [115]. Concurrently, other interorgan neuroimmune circuits also impact immunotherapy: ATF4–SLIT2–CGRP signaling in head and neck squamous cell carcinoma (HNSCC) and in a murine model of oral cancer (MOC) prompts cancer cells to activate nociceptive neurons, which in turn triggers a process of lymph node remodeling and immunosuppression via the action of CGRP and ultimately reduces the efficacy of immune checkpoint blockade; targeting this axis re‐activates immune activity and enhances checkpoint blockade responses [32]. There is also evidence of remote neural effects in SCLC which is a highly neuroendocrine lung cancer with a high tendency to spread to the brain [111, 143]. Recent transcriptomic and electrophysiological studies suggest that SCLC cells are able to interact functionally with central neurons, to cause multiplicative synaptic upscaling and to dysregulate the excitability of neurons, in part by using NMDA receptor‐dependent mechanisms [111]. Based on these findings, neuronal excitability could be a mechanism to consider when looking at neurological symptoms in SCLC brain involvement and may also be a direct cause of clinical cognitive deficits, but further validation is required [111, 144].

#### 3.3.2 Electrophysiological Integration in Gliomas

In contrast, high‐grade gliomas (HGGs) form a strong connection with neural tissue [145]. These tumors are not simply a mass effect on the brain; they actually become a part of the brain's neural networks, electrically. Functional synapses between glutamatergic neurons and glioma cells have been reported that involve AMPA receptors (AMPARs). During nervous system activity, glioma cells receive excitatory currents, and these currents directly stimulate the proliferation of the cells [72, 146]. The effect is multiplied by the fact that the glioma cells are electrically coupled by gap junctions to form a syncytium. Furthermore, the cleavage of neuroligin‐3 (NLGN3) is also associated with neural activity and promotes tumor growth. These mechanisms are all linked together in a feed‐forward fashion that allows the tumor to use the endogenous brain electrical signals to promote its growth [21, 147].

## 4 The Neuro‐Immune Interface in Tumors

### 4.1 Conceptual Framework of the Neuro‐Immune‐Tumor Microenvironment

The neural–epithelial interaction is not limited to direct contact, but has a significant impact on the host immune environment [148, 149]. This tripartite interaction is called the neuro–immune–tumor microenvironment [150], and nerve fibers are emerging as key players in tumor immunity [14, 80, 150, 151]. Sometimes neural networks do not directly stimulate cancer cells but indirectly promote tumor progression by inducing changes in the phenotype and activity of immune cells and in the tissue architecture [151, 152]. Advanced molecular technologies have substantially improved our understanding of this spatial and functional heterogeneity. Recent spatial transcriptomics and multiplex immunofluorescence studies have delineated the distribution of nerve fibers within human tumors, showing that sensory nerve endings are spatially associated with immune infiltrates and cancer‐associated fibroblasts (CAFs). These interactions may establish physical barriers that impair the formation of antitumor tertiary lymphoid structures (TLS) [88, 153].

Furthermore, single‐cell RNA sequencing (scRNA‐seq) has identified neuronally responsive immune subpopulations. For instance, recent scRNA‐seq analyses have identified specific “neuron‐like” macrophage subsets that express neural receptors, revealing dynamic transcriptional shifts in immune cells exposed to neural signals and their contribution to an immunosuppressive TME niche [154].

In lung cancer, including NSCLC and SCLC, recent scRNA‐seq atlases have systematically mapped the phenotypic spectrum of neuronally responsive immune infiltrates [33, 155]. Moreover, spatial transcriptomics [156, 157] has extended analyses beyond bulk tissue profiling [158], quantitatively demonstrating the single‐cell‐level proximity of sympathetic and sensory nerve endings, exhausted CD8^+^ T cells, and CAFs [159, 160, 161, 162, 163] (Figure 1).

### 4.2 Sympathetic Modulation of Tumor Immunity

The sympathetic nervous system suppresses antitumor immunity primarily through the release of catecholamines, including norepinephrine and epinephrine. Under chronic psychological stress or locally increased sympathetic tone, these neurotransmitters bind to β2‐adrenergic receptors (β2‐ARs), which are widely expressed on diverse immune cell populations [164]. This adrenergic signaling exerts potent immunosuppressive effects through several distinct mechanisms (Figure 1).

#### 4.2.1 T‐Cell Exhaustion and Trafficking

Catecholamines directly impair CD8+ T‐cell cytotoxicity by altering their metabolic fitness. Activation of β2‐ARs on CD8+ T cells reduces their glycolytic capacity, thereby suppressing the production of effector molecules such as IFN‐γ and granzyme B [165, 166]. Furthermore, sympathetic innervation of the lymphatic system strongly regulates immune cell trafficking. β‐Adrenergic signaling modulates T‐cell trafficking in lymphoid tissues and can alter leukocyte migration dynamics [165, 167].

#### 4.2.2 Expansion of Suppressive Myeloid Cells

Sympathetic signaling promotes the accumulation of immunosuppressive cell populations. Catecholamine‐induced β2‐AR activation enhances the survival of myeloid‐derived suppressor cells (MDSCs) through STAT3 phosphorylation, upregulates PD‐L1 expression, and shifts their metabolism toward fatty acid oxidation, thereby increasing their immunosuppressive capacity [168, 169]. Additionally, catecholamine signaling polarizes TAMs toward an M2‐like, pro‐angiogenic phenotype by increasing the secretion of VEGF and IL‐6 [169]. By reprogramming these immune components, sympathetic nerves shield tumors from immune surveillance.

### 4.3 Parasympathetic and Sensory Modulation of the Immune Landscape

#### 4.3.1 Parasympathetic (Cholinergic) Regulation

In contrast to the sympathetic axis, parasympathetic, or vagal, signaling exerts complex and context‐dependent effects on tumor immunity. The classical “cholinergic anti‐inflammatory pathway” involves acetylcholine (ACh) binding to α7 nicotinic acetylcholine receptors (α7nAChRs) on macrophages, thereby suppressing NF‐κB activation and inhibiting TNF‐α release [165, 170]. In certain oncological contexts, however, vagal stimulation can exert pro‐immune effects. For instance, efferent vagal activity stimulates memory CD4+ T cells to secrete the anti‐inflammatory peptide trefoil factor 2 (TFF2) in the spleen and TME [171]. This neurally induced TFF2 binds to CXCR4 receptors on immature myeloid cells and selectively limits the pathological expansion of myeloid‐derived suppressor cells (MDSCs), thereby reversing systemic immunosuppression and enhancing cellular antitumor immunity in colorectal and pancreatic cancers [170, 171] (Figure 1).

#### 4.3.2 Sensory Nerves and Neuropeptides (Substance P and CGRP)

Sensory nociceptors innervating the TME actively shape the immune landscape by releasing potent neuropeptides, thereby driving a sustained state of neurogenic inflammation [87, 136]. Two critical sensory mediators, substance P (SP) and calcitonin gene‐related peptide (CGRP), have emerged as key regulators of tumor immune evasion.

By binding to the neurokinin‐1 receptor (NK‐1R), SP activates NF‐κB and MAPK signaling pathways in breast and pancreatic cancers [84, 172, 173]. This signaling not only promotes neurogenic inflammation [174, 175, 176] but also confers anoikis resistance on cancer cells and enhances their motility [84, 85, 172]. Within the immune context, chronic SP–NK1R signaling upregulates IL‐10 and proangiogenic factors, shifting tumor‐associated macrophages toward an immunosuppressive, protumoral M2‐like state while suppressing cytotoxic immune responses [128, 177].

CGRP also acts as an important immunomodulator through receptor complexes containing receptor activity‐modifying protein 1 (RAMP1) and CALCRL on cytotoxic CD8+ T cells [87, 148]. This CGRP–RAMP1 signaling axis attenuates T‐cell receptor (TCR) activation through the Gαs–cAMP–PKA pathway, driving profound CD8+ T‐cell exhaustion [87, 178]. This molecular event induces marked upregulation of immune checkpoints, including PD‐1, TIM3, and LAG3, on RAMP1+ CD8+ T cells, and impairs the formation of tertiary lymphoid structures [87]. Consequently, neuropeptide‐driven exhaustion helps establish a tolerogenic niche that limits immune‐mediated tumor clearance and contributes to resistance to immune checkpoint blockade therapies [87, 148]. Thus, pharmacological blockade of sensory neuropeptide signaling, for example with clinically approved CGRP receptor antagonists such as Rimegepant, is emerging as a translational strategy to enhance cancer immunotherapy [87, 179] (Figure 1).

### 4.4 Bidirectional Neuro‐Immune Crosstalk

In the TME, immune cells are not only subject to neuroregulation but also downregulate neural structure and function through the secretion of factors, exosomes, and direct interactions, forming a critical “immune‐to‐neural” feedback loop.

#### 4.4.1 Immune Cells Secrete Neurotrophic Factors That Drive Tumor Innervation

Brain‐derived neurotrophic factor (BDNF): BDNF is often upregulated in response to chronic adrenergic stress [95], and binds to the TrkB receptor to suppress anoikis [94] and promote epithelial–mesenchymal transition (EMT) [180]. This signaling loop is closely associated with tumor cell dissemination and distant metastatic colonization [94, 180]. Bone marrow‐derived macrophages are the primary source of BDNF in triple‐negative breast cancer; the BDNF they secrete is both necessary and sufficient for nerve fiber invasion and growth—removing macrophages or inhibiting BDNF signaling significantly reduces nerve invasion and tumor progression [181]. In a lung adenocarcinoma model, tumor‐associated macrophages directly promote tumor neurogenesis through macrophage‐to‐neuron transition (MNT) and are associated with cancer pain behavior; single‐cell transcriptomic analysis confirms that Smad3 is a key regulator of this transition [154].

#### 4.4.2 Immune Mediators Regulate the Activity of Pain Neurons and Cancer Pain

Tumor‐infiltrating immune cells can directly regulate local neuronal activity, particularly in pain‐transmitting neurons [148]. In a mouse model of bone cancer pain, tumor burden significantly increased spinal cord TNF‐α mRNA levels, accompanied by spontaneous paw withdrawal, mechanical hyperalgesia, and thermal hyperalgesia; the TNF‐α antagonist etanercept effectively reduced spinal cord inflammatory cytokine levels and alleviated pain‐related behaviors, confirming the sensitizing effect of immunogenic TNF‐α on pain pathways [182]. In head and neck squamous cell carcinoma, cancer cells treated with norepinephrine secrete higher levels of TNF‐α, a factor that enhances calcium transients in trigeminal neurons [183]; systemic administration of the β‐adrenergic blocker propranolol or chemical parasympathetic denervation significantly reduces pain‐related behavior associated with oral cancer [184].

#### 4.4.3 Immune Cell Exosomes and Soluble Factors Reshape the Neural Microenvironment

Exosomes and soluble factors derived from immune cells work synergistically through various mechanisms to reshape the tumor microenvironment of the central nervous system.

##### 4.4.3.1 Regulation and Carrier Functions of Immune Cell Exosomes

Exosomes, as key mediators of intercellular communication, play a dual role in the remodeling of the immune microenvironment in brain tumors. Exosomes derived from genetically engineered M1 macrophages, loaded with the histone deacetylase inhibitor palbistat and small interfering RNA (siSTAT3) targeting signal transduction and transcription activator 3 (STAT3), can cross the blood–brain barrier to precisely target glioblastoma cells, significantly inhibiting tumor proliferation and invasion, while simultaneously reducing astrocyte reactivity and M2 macrophage infiltration, thereby achieving reprogramming of the tumor microenvironment [185]. In addition, engineered exosomes derived from immune cells such as dendritic cells, M1 macrophages, and neutrophils can regulate the functions of various immune cell types—including T cells, B cells, NK cells, and myeloid suppressor cells—by carrying noncoding RNA (such as miRNA), thereby playing a key regulatory role in tumor initiation and progression [186, 187]. Due to their low immunogenicity, inherent homing ability, and capacity to cross the blood–brain barrier, immune cell exosomes have emerged as highly promising drug and gene delivery vehicles and cancer vaccine platforms, demonstrating clear potential for precision intervention in brain tumors such as glioblastoma [188].

##### 4.4.3.2 Soluble Factor‐Mediated Neuro‐Immune Remodeling

Soluble factors secreted by immune cells form a complex cytokine network in the tumor microenvironment, driving immune suppression and the malignant phenotype of tumors. In glioblastoma, TGF‐β, IL‐10, IL‐6, IL‐1β, and the chemokine CXCL12, among others, can suppress the antitumor functions of T cells, NK cells, and dendritic cells, promote the recruitment of regulatory immune cells, and directly enhance tumor cell proliferation, invasion, and resistance to treatment. Under conditions of chronic inflammation, type I and type II interferons can also induce immune exhaustion and upregulation of immune checkpoint molecules [189].

Furthermore, as a key immunoregulatory molecule, galectin‐9 is highly expressed by microglia and macrophages in the brain tumor microenvironment under the regulation of hypoxia‐inducible factor‐2α (HIF‐2α), and its expression levels directly influence the functional state of myeloid cells; blocking galectin‐9 inhibits glioma growth and reshapes the activity of T cells and myeloid cells, suggesting that it is an important target for immunotherapy [190].

Interactions within the neuro–immune–tumor axis should also not be overlooked. Neurons can promote an immune‐rejection phenotype in gliomas through glutamate release mediated by AMPARs; Combining AMPAR antagonists with immune checkpoint inhibitors, or integrating them with neuromodulation techniques such as repetitive transcranial magnetic stimulation (rTMS) and deep brain stimulation (DBS), may offer a way to modulate this axis and improve the efficacy of immunotherapy [72, 191, 192].

Brain metastases exhibit unique immunosuppressive characteristics. In brain metastases from lung cancer, the expression levels of T‐cell infiltration density, cytolytic markers (such as granzyme B), and co‐inhibitory receptors (PD‐1, TIM‐3, LAG‐3) are all significantly lower than in the primary tumor; however, high CD3^+^ T‐cell infiltration and high TIM‐3/LAG‐3 expression are associated with longer overall survival in patients, suggesting that the microenvironment of brain metastases exhibits unique spatiotemporal heterogeneity, which supports the development of targeted immunotherapy strategies for intracranial tumors [193].

Immune cell exosomes and soluble factors act synergistically in the neural microenvironment, jointly promoting M2 macrophage polarization, regulatory T cell expansion, and immune cell exhaustion, thereby exacerbating tumor progression and treatment resistance [194, 195, 196]. Engineering strategies targeting this complex network have achieved breakthroughs. For example, by engineering hematopoietic stem cells using lentiviral vectors to enable the specific and controlled release of IFN‐α or IL‐12 at the tumor site, it is possible to achieve spatiotemporal controlled reprogramming of the glioblastoma immune microenvironment, inducing a pro‐inflammatory state while avoiding systemic toxicity. Such strategies have already entered the clinical trial phase [197].

However, clinical translation still faces challenges. Although exosome‐based delivery systems offer significant advantages in terms of targeted delivery and biocompatibility, issues related to their large‐scale production, purification, and standardization remain to be addressed. Future research should combine multi‐omics analyses to identify key regulatory factors (such as miR‐3591‐3p and Galectin‐9) and develop multipathway synergistic intervention strategies to address the spatiotemporal heterogeneity of the microenvironment [198, 199, 200, 201].

## 5 Therapeutic Opportunities and Challenges

**FIGURE 3:**
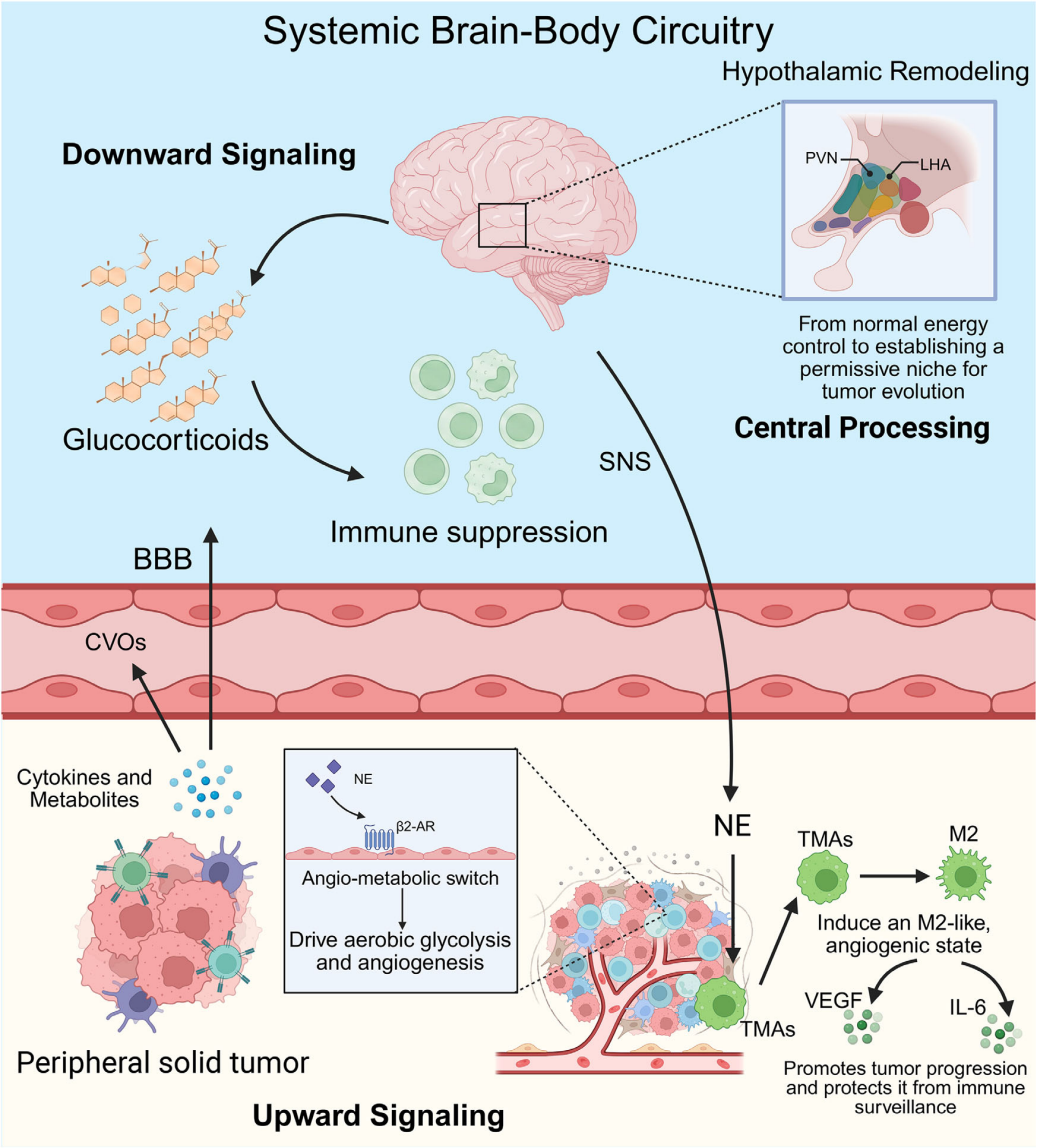
Clinical translation pathway in cancer neuroscience: from targetable neural vulnerabilities to the management of neurotoxicity. (A) Shift in therapeutic paradigm. The field is evolving from crude structural denervation (left), which causes systemic off‐target dysregulation, toward precision neural reprogramming (right), which selectively intercepts specific neuro‐oncological signaling nodes while preserving physiological neural function. (B) Histology‐specific targeted strategies. In non–small cell lung cancer (NSCLC, top), stress‐induced sympathetic signaling impairs CD8+ T cell function via β2‐adrenergic receptors (β2‐AR). Combining nonselective β‐blockers with immune checkpoint inhibitors (anti‐PD‐1) synergistically reinvigorates antitumor immunity. Meanwhile, CNS‐penetrant targeted therapies effectively disrupt intracranial metastatic niches. In small cell lung cancer (SCLC) and gliomas, malignant cells form functional glutamatergic pseudo‐synapses with host neurons. Pharmacological blockade of NMDAR/AMPAR, ADAM10‐mediated NLGN3 cleavage (INCB7839), or Notch2/3‐driven lineage plasticity (tarextumab) short‐circuits these tumor–neural networks. (C) The caveat is neurotoxicity. Conventional therapies inflict severe collateral damage. Chemotherapeutic agents drive chemotherapy‐induced peripheral neuropathy (CIPN) through mitochondrial DNA damage and axonal degeneration in dorsal root ganglia. Systemic agents like methotrexate (MTX) cross the blood–brain barrier (BBB), triggering microglial activation and myelin dysregulation, leading to chemotherapy‐related cognitive impairment (CRCI). Future strategies must prioritize neuroprotective buffering alongside neural‐targeted therapies.

There is an emerging paradigm shift in cancer biology: tumors are increasingly recognized not merely as localized masses of mutated cells, but as deeply innervated “organs” that actively exploit the host nervous system [1, 202]. This knowledge is a paradigm shift in therapeutic approach [1, 129]. Initial preclinical experiments have also shown that surgical denervation can inhibit tumor growth; however, physically removing main nerves is not feasible and has a catastrophic systemic effect [9, 203, 204]. The use of translation strategies is more focused and is the current trend [136, 205]. Current strategies do not involve ablating nerves, but rather target specific molecular cross‐talk known to occur between tumors and neurons, and combine these neuromodulatory therapies with existing clinical practices [136, 206] (Table 2; Figure 3).

**TABLE 2:** Summary of clinical trials targeting the nervous system and emerging pharmacological strategies targeting neural‐tumor crosstalk.

Therapeutic strategy and target axis	Intervention and setting	Targeted malignancy	Clinical trial status (ID/phase)	Key objectives and clinical evidence	Representative references
**Immunotherapy augmentation** •*Pathway*: β‐adrenergic signaling •*Target*: β‐adrenergic receptors (β‐AR)	**Propranolol** •Combined with pembrolizumab	Metastatic melanoma	**NCT03384836** •Phase Ib/II •Phase Ib completed	•Objective: To determine the recommended phase II dose, safety, tolerability, and preliminary antitumor activity •Findings: No dose‐limiting toxicities observed; ORR was 78%; 30 mg twice daily recommended for phase II (Early‐phase clinical evidence)	[207, 208, 209]
**Immunotherapy modulation** •*Pathway*: β‐adrenergic signaling •*Target*: β‐AR	**β‐blockers** •Concurrent use with immune checkpoint inhibitors	Advanced non–small cell lung cancer	**N/A** •Retrospective evidence only	• Objective: To evaluate whether concomitant β‐blocker use is associated with improved outcomes during ICI therapy •Findings: Retrospective studies showed heterogeneous results with no definitive prospective validation (retrospective clinical evidence)	[210, 211]
**Perioperative neuroimmune modulation** •*Pathway*: β‐adrenergic and inflammatory stress signaling •*Target*: β‐AR/stress‐associated inflammatory pathways	**Propranolol ± etodolac** •Perioperative administration	Breast cancer	**N/A** •Phase II •Completed	•Objective: To reduce perioperative prometastatic and inflammatory biomarkers associated with surgical stress •Findings: Randomized phase II studies showed favorable modulation of metastatic biomarkers and stress‐related transcriptional programs (early‐phase translational clinical evidence)	[212, 213]
**Perioperative autonomic modulation** •Pathway: β‐adrenergic signaling •*Target*: β‐AR	**Propranolol** •Preoperative/perioperative intervention	Pancreatic ductal adenocarcinoma	**NCT06145074** •Phase II pilot •Recruiting/ongoing	•Objective: To assess safety, feasibility, and biological impact of preoperative propranolol. •Findings: No mature efficacy results available; rationale supported by strong preclinical/translational evidence (prospective pilot/translational evidence)	[56]
**(Local denervation strategy** •Pathway: Autonomic nerve innervation •Target: Intraprostatic neural input	**Botulinum toxin A** •Local injection before prostatectomy	Prostate cancer	**NCT01520441** •Pilot/early‐phase •Withdrawn	•Objective: To examine biological endpoints associated with intraprostatic denervation in localized prostate cancer. •Findings: No efficacy conclusions can be drawn, as pilot study was withdrawn (Pilot/feasibility concept)	[5]
**(Synaptic dependency targeting** •Pathway: ADAM10/NLGN3 axis •Target: ADAM10/NLGN3 shedding	**INCB007839 (INCB7839)** •Monotherapy	Pediatric high‐grade glioma	**NCT04295759** •Phase I •Closed/early‐phase reported	•Objective: To determine safety, tolerability, and pediatric dosing of ADAM10 inhibition •Findings: Early clinical reporting supports feasibility and safety; antitumor efficacy remains to be established (Phase I clinical with strong preclinical rationale)	[214, 215];
**Synaptic dependency targeting** •Pathway: AMPA receptor‐mediated neuron–glioma signaling •Target: AMPA receptor	**Perampanel** •Around surgery/translational antitumor‐seizure setting	Progressive or recurrent glioblastoma	**NCT03636958** • Phase IIa • Ongoing	•Objective: To evaluate effects on peritumoral hyperexcitability, neuron–tumor synaptic connectivity, and progression •Findings: Trial protocol published; definitive efficacy results pending (early‐phase clinical with strong mechanistic rationale)	[71, 72, 216]
**Glutamatergic circuit targeting** •Pathway: NMDA receptor signaling •Target: NMDA receptor	**Memantine** •Radiation‐associated neuroprotection (indirect tumor relevance)	Primary CNS tumors	**NCT04939597** •Phase III •Ongoing	•Objective: To determine whether memantine preserves cognition during cranial radiotherapy •Findings: Clinical evidence currently supports neuroprotection rather than direct antitumor targeting (indirect clinical support with preclinical rationale)	[73]
**(Network disconnection strategy** •Pathway: Gap junction/tumor microtube network •Target: Connexin‐mediated intercellular coupling	**Meclofenamate + temozolomide** •Recurrent setting	MGMT‐methylated recurrent glioblastoma	**EudraCT 2021‐000708‐39** •Phase I/II •Registered/protocol published	•Objective: To assess toxicity and preliminary efficacy of meclofenamate added to second‐line temozolomide •Findings: Protocol and rationale published; mature efficacy data not yet available (early‐phase clinical with network biology rationale)	[217, 218]
**Neurotrophic signaling/supportive care** •Pathway: NGF signaling •Target: Nerve growth factor (NGF)	**Tanezumab** •Background opioid therapy permitted	Cancer pain due to bone metastasis	**NCT02609828** •Phase III •Completed	•Objective: To evaluate efficacy and safety of anti‐NGF therapy for cancer bone pain •Findings: Placebo‐controlled study demonstrated analgesic benefit; regarded as supportive care rather than direct anticancer therapy Phase III supportive‐care trial)	[219, 220]
**Cholinergic pathway modulation** •Pathway: Muscarinic signaling •Target: M3R / M1R	Darifenacin, atropine (or other cholinergic modulators) •Preclinical concept	Gastric, colorectal, and pancreatic cancer	**N/A** •Preclinical	•Objective: To modulate cholinergic circuits influencing tumor stemness, proliferation, or immune remodeling • Findings: Strong mechanistic evidence exists, but representative oncology trials have not yet been established (preclinical evidence)	[9, 54, 56]
**Sensory nerve–immune axis targeting** •Pathway: CGRP‐mediated immune suppression •Target: CGRP receptor/RAMP1	**Rimegepant (or other CGRP receptor antagonists)** •Potential combination with anti‐PD‐1	Melanoma, oral squamous cell carcinoma	**N/A** •Preclinical	•Objective: To reverse sensory neuron‐induced CD8+ T‐cell dysfunction and tumor‐protective autophagy •Findings: Strong preclinical evidence supports immunologic and tumor‐control benefits without established trials yet (preclinical evidence)	[87, 88, 221, 222]
**Neuroimmune modulation** •Pathway: Substance P/TACR1 signaling •Target: NK1R/TACR1	**Aprepitant** •Repurposing/adjunctive concept	Breast cancer, pancreatic cancer	**N/A** •Preclinical/translational	•Objective: To interrupt substance P‐driven invasion, neuroinflammation, and TME remodeling •Findings: Compelling preclinical rationale exists, but formal oncology trial evidence remains limited (preclinical/translational evidence)	[84, 86]
**CNS‐penetrant targeted therapy** •Pathway: ALK signaling with CNS tropism •Target: ALK	**Ensartinib** •Monotherapy comparator trial	ALK‐positive non–small cell lung cancer	**eXalt3** •Phase III •Completed	•Objective: To compare ensartinib with crizotinib, including intracranial disease control •Findings: Ensartinib demonstrated superior systemic and intracranial efficacy compared with crizotinib (Phase III evidence)	[223]
**Developmental lineage/neuroendocrine plasticity targeting** •Pathway: Notch signaling •Arget: Notch2/3	**Tarextumab** •Combined with platinum etoposide	Extensive‐stage small cell lung cancer	**N/A** •Phase Ib •Completed early‐phase exploration	•Objective: To assess safety and preliminary efficacy of Notch pathway blockade in combination with chemotherapy •Findings: Exploratory early‐phase data reported, but strategy did not become practice‐changing (preclinical plus early clinical evidence)	[224]
**Neuromodulatory targeted therapy** •Pathway: Dopaminergic signaling •Target: DRD2/DRD3	**ONC201 (dordaviprone)** •Monotherapy	H3K27M‐mutant diffuse midline glioma, spinal cord glioma, neuroendocrine tumors	**NCT02525692; NCT03295396; NCT03034200** •Phase I/II •Mixed status	•Objective: To evaluate antitumor activity and disease control in CNS and neuroendocrine malignancies •Findings: Clinical activity reported in recurrent H3K27M‐mutant diffuse midline glioma; broader development remains indication‐specific (Phase I/II clinical evidence)	[225, 226, 227]

### 5.1 Targeting Neural Signaling Pathway

In recent years, therapeutic strategies targeting key signaling pathways involved in neuro‐tumor interactions have increasingly emerged as a key approach to overcoming treatment resistance and enhancing antitumor efficacy. Based on an in‐depth understanding of the mechanisms underlying the abnormal activation of pathways such as Gi/o‐GPCR, PI3K/AKT, Src, and Erk under neurogenic regulation, various small‐molecule inhibitors, GPCR modulators, and combination therapies have demonstrated significant potential in preclinical models. Furthermore, integrating biomarker‐guided, personalized treatment approaches is gradually driving this field from mechanistic exploration toward clinical translation.

#### 5.1.1 Evidence of the Efficacy of Small‐Molecule Inhibitors in Preclinical Models

Multiple studies have shown that small‐molecule inhibitors targeting key signaling nodes such as PI3K/AKT, Src, and MEK exhibit clear antitumor activity in solid tumor models driven by neuro‐tumor interactions. In HER2‐positive breast cancer, Gi/o‐GPCR signaling promotes the maintenance of tumor stem cells and resistance to HER2‐targeted therapy through the PI3K/AKT and Src pathways; the combined use of diphtheria toxin (PTx) to dissociate Gi/o‐GPCRs from G proteins, followed by treatment with PI3K or Src inhibitors, can significantly inhibit cancer cell proliferation, migration, and tumor formation and metastasis in vivo [228]. Similarly, in GBM, neuron‐activity‐dependent paracrine signaling and synapse‐like connections activate the Akt/Erk pathway; the use of MEK or AKT inhibitors can effectively block this tumor‐promoting axis [229]. In addition, low‐dose MEK, AKT, or Notch inhibitors can induce pericyte maturation, improve oxygenation in the tumor microenvironment, and enhance T‐cell infiltration, thereby enhancing the efficacy of adoptive T‐cell therapy in melanoma models [230]. Taken together, this evidence supports the key role of small‐molecule inhibitors in disrupting neurogenic signal transduction.

##### 5.1.1.1 GPCR modulators

G protein‐coupled receptors (GPCRs), as the primary effectors of neurotransmitters, chemokines, and neurotrophic factors, play a pivotal role in neuro‐tumor crosstalk. In glioblastoma, tumor cells hijack glutamatergic and GABAergic synaptic signaling to mediate proliferation and survival through metabolic and ionotropic glutamate receptors (such as mGluR and NMDAR); Antagonists or allosteric modulators targeting these GPCR subtypes hold promise for blocking abnormal neuro‐tumor communication. In HER2‐positive breast cancer, abnormal Gi/o‐GPCR expression is driven by HER2 overexpression, and its downstream signaling promotes treatment resistance; experiments have shown that PTx‐mediated Gi/o decoupling can reverse this phenotype, suggesting that the development of selective Gi/o‐GPCR antagonists holds therapeutic value [228] In addition, neuropeptide P released by sensory nerve fibers within tumors promotes proliferation and migration by activating NK1R (a GPCR) on tumor cells, and NK1R antagonists have demonstrated cross‐cancer intervention potential in various tumor types [231, 232, 233, 234]. In summary, the use of GPCR modulators in glioblastoma and breast cancer is not only mechanistically sound but also has the potential for clinical translation [235].

### 5.2 Combination With Immunotherapy

Given that inhibiting a single pathway can easily trigger feedback activation and bypass compensation, a combined strategy has become key to overcoming therapeutic bottlenecks. On the one hand, targeting neuro‐tumor interaction pathways can reshape the immune microenvironment and enhance the efficacy of ICIs. For example, tumor‐activated Schwann cells produce prostaglandin E via the TGF‐β‐MAPK/ERK axis, inducing T‐cell exhaustion (manifested as upregulation of PD‐1 and CD73), whereas MAPK/ERK kinase inhibitors can block this process and restore T‐cell function [236]. On the other hand, high expression of neural signals is associated with immune rejection, suggesting that inhibiting neurogenic pathways may transform “cold tumors” into “hot tumors” [158]. In addition, the SHP2 inhibitor PF‐07284892, when combined with previously ineffective targeted therapies, induced rapid ctDNA responses and prolonged the duration of clinical benefit in models such as ALK‐fusion‐positive lung cancer [237]. These strategies highlight the advantages of multimodal synergistic interventions in overcoming neurally mediated drug resistance.

### 5.3 Disease‐Specific Considerations

#### 5.3.1 Calming the Microenvironment: Neuromodulation in NSCLC

Abnormal activity of the autonomic nervous system (ANS) is an active participant in the tumor immune microenvironment (TIME) of NSCLC [238, 239]. Chronic stress and subsequent hyperactive sympathetic nervous system [80, 240] result in a higher concentration of neurotransmitters such as norepinephrine in the tumor microenvironment [80, 241]. Kokolus et al. and Bucsek et al. demonstrated that sympathetic activation may disrupt the CD8+ T‐cell effector functions and the cross‐presentation of antigens from the tumor by dendritic cells [166, 242]. Simultaneously, adrenergic signaling triggers an “angio‐metabolic switch” in tumor cells, resulting in higher glycolysis and higher PD‐L1 levels by means of the PI3K/Akt/mTOR pathway [128] (Figure 1).

From a translational clinical perspective, neural blockade may be combined with immune checkpoint inhibitors (ICIs). In preclinical orthotopic models, propranolol, a nonselective β‐blocker, has been shown to attenuate sympathetic overactivation, reduce the tumor immunosuppressive barrier, and enhance T‐cell‐mediated cytotoxicity [242]. These preclinical findings are supported by recent large‐scale retrospective cohort studies, including the study by Cortellini et al. [243], which reported improved overall survival and objective response rates in NSCLC and melanoma patients receiving anti‐PD‐1 therapy together with β‐blockers.

Recent retrospective cohort studies and prospective clinical trials have further supported the translational relevance of β‐blockade in oncology [243, 244]. Extensive epidemiological and real‐world evidence also suggests that concurrent beta‐blocker use may be associated with prolonged progression‐free survival (PFS) and overall survival (OS) in patients with metastatic NSCLC receiving ICIs [207, 209, 210, 211, 245] (Table 2; Figure 3).

#### 5.3.2 SCLC: Rewiring the “Pseudo‐Neuron”

SCLC has distinct biological features compared with other types of lung cancer [107, 246]. Because of its intrinsic neuroendocrine (NE) lineage and strong neurotropism, SCLC can recruit adjacent nerves and also functionally mimic neural cells [247]. One major contributor to treatment failure in SCLC is its pronounced intratumoral heterogeneity. Lim et al. (Nature) showed that Notch signaling can act as a molecular fate switch, enabling classic neuroendocrine cells (ASCL1+) to transition into a chemoresistant, nonneuroendocrine state (REST+) [224]. These non‐NE cells act in a paracrine manner by providing neurotrophic factors that support the aggressive NE subpopulation [224] (Figure 3).

Knocking out this Notch‐driven fate transition could be a therapeutic approach. In patient‐derived xenografts (PDXs) and genetically engineered mouse models (GEMMs), researchers demonstrated that combining standard platinum/etoposide chemotherapy with a Notch2/3 antagonist, like tarextumab, can effectively block this transition and significantly delay SCLC relapse [110, 224] (Figure 3). It may be possible to inhibit SCLC proliferation and central nervous system metastasis [73] by targeting these electrochemical vulnerabilities with specific NMDA receptor antagonists (like memantine), or voltage‐gated channel blockers (Figure 1).

#### 5.3.3 Reprogramming Neuro–Tumor Communication in GBM: A New Therapeutic Paradigm for Blocking Neurogenic Growth Signals

Recent conceptual models have focused not on the general ablation of neural tissue, but rather on precise “reprogramming”, as mentioned by Wang et al. [14, 248]. Selective inhibition of paracrine or synaptic signals, on which tumors rely, is possible rather than systemic targeting of the nervous system.

HGG are a representative example of this paradigm. Venkatesh et al. [215] showed that these tumors make use of the synaptic adhesion molecule neuroligin‐3 (NLGN3) that is shed by neighboring active neurons. Instead, a specific inhibitor of ADAM10, INCB7839, blocks shedding of NLGN3 into the tumor microenvironment [72]. This is a good way to inhibit the tumor's ability to receive neuronal growth signals without impairing brain function and is now under study in clinical trials in children. Similarly, the use of dopamine receptor D2 (DRD2) antagonists, like ONC201 (imipridone), for clinical or preclinical treatment of neuroendocrine and glial tumors has been shown to effectively reprogram the aberrant transcriptional landscape of the tumors, a process rather than destroying neurons, and thus, target cancer by modulating signaling interactions [226, 249] (Table 2).

### 5.4 Clinical Landscape, Biomarkers, and Safety

#### 5.4.1 The Future: Reprogramming the Neural‐Tumor Dialogue

In the future, “reprogramming the neuro‐tumor dialogue” should not be limited to nonselective denervation but should evolve into spatiotemporally precise combination therapies based on the tumor's neural phenotype. Existing preliminary studies indicate that ablation of autonomic or sensory nerves can delay the onset and progression of prostate, gastric, and pancreatic cancers [5, 9, 25]; blocking β‐adrenergic signaling, the cholinergic–NGF/Trk feedback loop, and nerve‐activity‐dependent NLGN3/ADAM10 and AMPA receptor signaling may disrupt nerve‐driven tumor proliferation, vascular–metabolic adaptation, and neural circuit integration [7, 54, 71, 215].

At the same time, β2‐adrenergic signaling can suppress the effector function and metabolic reprogramming of CD8^+^ T cells, suggesting that neurotargeted therapy can enhance the efficacy of immune checkpoint blockade by reversing neurogenic immunosuppression [166, 208]; a Phase I study of propranolol in combination with pembrolizumab and a randomized perioperative trial in breast cancer have also preliminarily demonstrated the clinical feasibility of this strategy, though these findings are not yet sufficient to establish a survival benefit [207, 213].

Therefore, in the future, we should rely on spatial multi‐omics, neural tracing, electrophysiology, and patient‐derived models to identify neural subtypes, neurotransmitter–receptor networks, and immunometabolic states; establish neurobiomarkers for patient stratification and treatment response monitoring; and prioritize the development of localized, reversible neuromodulation approaches that preserve organ function, combining them with immunotherapy, targeted therapy, or chemoradiotherapy in a mechanism‐driven manner; At the same time, prospective randomized studies are needed to systematically evaluate neural plasticity, compensatory redirection, and long‐term neurotoxicity, thereby advancing interventions targeting the neuro–tumor dialogue from proof‐of‐concept to truly implementable precision cancer therapies.

#### 5.4.2 The Caveat of Neurotoxicity

While these are good signs, there is one significant limitation, namely, that the traditional therapies are quite neurotoxic [19, 250]. Platinum agents and taxanes are often associated with dose‐limiting chemotherapy‐induced peripheral neuropathy (CIPN), and this is thought to be caused by direct damage to mitochondrial DNA in dorsal root ganglia and persistent activation of small‐fiber neuroinflammation [250, 251, 252, 253, 254, 255]. During treatment with systemic agents like methotrexate, microglial activation occurs, and myelin is disregarded in a long‐lasting manner, leading to the debilitating chemotherapy‐induced cognitive impairment (CRCI) commonly referred to as chemobrain [19, 256, 257]. Future neuro‐oncological therapies should be in a delicate balance: to target selectively circuits supporting the tumor without disturbing the entire neural homeostasis [19, 250, 256, 258].

Recent mechanistic breakthroughs have clarified the complex pathophysiology underlying CIPN and CRCI. State‐of‐the‐art models indicate that taxane‐ and platinum‐induced neurotoxicity is largely driven by sustained neuroinflammation, reactive astrogliosis, and pathological microglial activation in the dorsal root ganglia and CNS [19, 259, 260, 261, 262]. Furthermore, contemporary multi‐omics studies have identified metabolic derangements and mitochondrial DNA damage [213] signatures as key drivers of these side effects, prompting the development of novel neuroprotective adjuncts that preserve chemotherapeutic efficacy [263, 264, 265, 266] (Table 2; Figure 3).

#### 5.4.3 Personalized Therapy and Biomarker‐Guided Treatment Based on Pathway Activity

In HER2‐positive breast cancer, high expression of Gi/o‐GPCRs can serve as a screening marker for combination therapy with PI3K/Src inhibitors [228]; in gliomas, single‐cell RNA sequencing has identified dynamic signaling modules associated with neuronal interactions, providing a basis for molecular subtyping in the application of small‐molecule inhibitors [229]. In the future, it will be necessary to integrate multi‐omics data, functional imaging (such as molecular probe‐based assessment of pathway activity [267]), and liquid biopsy (such as dynamic monitoring of ctDNA [237]) to establish a comprehensive biomarker system that can guide personalized treatment decisions based on the activity of neuro–tumor interaction pathways.

## 6 Conclusions

### 6.1 Summary of Overarching Principles

Tumors have long been considered as aggregates of genetically altered cells that act independently of the rest of the body. But this reductionist approach has been replaced by a more systemic approach. New findings in cancer neuroscience suggest that tumors are a pseudo‐organ, or complex whole, which hijacks and shapes the surrounding neural circuits for survival, for immune escape, and for metastatic spread [21, 165]. Thus, the neuro–cancer axis has gone from a peripheral phenomenon to a basic characteristic of malignancy [14].

This conceptual shift also requires a reconsideration of therapeutic strategies. Early preclinical studies largely relied on radical denervation through surgical or chemical approaches to interrupt the neural inputs supporting tumor growth. However, such widespread denervation lacks precision and may produce substantial off‐target effects, including disruption of metabolic and immune homeostasis [203]. Current research is therefore moving away from macroscopic neural ablation toward precision neural reprogramming [248]. Rather than eliminating entire neural networks, this approach aims to selectively intercept pathological signals at the tumor–nerve interface [248]. Existing models suggest that uncoupling the multicellular networks involved in neural–tumor communication may effectively suppress pro‐tumor neural crosstalk [268]. Potential strategies include targeting the ADAM10/NLGN3 axis [215], disrupting glutamatergic pseudo‐synapses [71, 73], and inhibiting neurotrophic pathways such as NGF/TrkA and BDNF/TrkB [269, 270]. Importantly, these precision approaches may better preserve normal electrophysiological functions in the host [72, 268].

A further overarching principle is that therapeutic disruption of tumor–nerve communication must be balanced against neurological safety. Conventional cancer treatments, including chemotherapy, radiotherapy, and immunotherapy, already impose a substantial neurological burden, which may manifest as chemotherapy‐induced peripheral neuropathy or cancer‐related cognitive impairment. These effects have been associated with neuroinflammation and impaired myelin homeostasis [19, 271]. Therefore, new therapies targeting neural–tumor communication should not introduce additional neurological toxicity. A more practical strategy may be to inhibit paracrine neurotrophic signals that facilitate tumor growth while sparing, or potentially protecting, neurons, astrocytes, and oligodendrocytes from systemic treatment‐related damage [2].

### 6.2 Top 3 Unanswered Questions in Cancer Neuroscience

#### 6.2.1 The Mechanisms Underlying the Integration of Neuro–Immune–Metabolic Pathways in the Tumor Microenvironment Remain Unclear

Although a growing body of evidence suggests that tumor‐associated neurons can modulate the state of immune cells through neurotransmitters, neuropeptides, and electrochemical signals, and that nutritional competition and immunosuppressive metabolites in the tumor microenvironment can further limit antitumor immunity, the so‐called “neuro–immune–metabolic pathway” is currently based primarily on evidence of pairwise interactions between neuro–tumor, neuro–immune, and metabolic–immune systems, and has not yet been established as a systematically validated, unified mechanistic framework [123, 136, 148, 272].

In particular, it remains unclear how different neural subtypes and their activity patterns synchronize and coordinate tumor cell metabolic adaptation, myeloid cell polarization, and T‐cell dysfunction within specific temporal windows and spatial niches through specific “neurotransmitter or neuropeptide–receptor–intracellular signaling–metabolic enzyme or metabolite” cascades; Most of the existing mechanistic evidence stems from a single cancer type and preclinical models; although a few studies have identified specific pathways through which neural signaling links tumor metabolic alterations to immune suppression, their tumor‐type specificity and generalizability remain to be verified [47, 148].

Therefore, future research should integrate circuit‐specific manipulation, single‐cell and spatial multi‐omics, stable isotope metabolic tracing, in vivo imaging, and neuro‐tumor organoid models to analyze the aforementioned causal chains from three dimensions—cell type, spatial structure, and disease progression—and to clarify the hierarchical relationships between local neural innervation and stress, circadian rhythms, and microbiota‐related neuroendocrine signals. Only on this basis can a reliable neuro‐immune‐metabolic biomarker system be established, enabling a rational assessment of the combined value of neurotargeted interventions with immune checkpoint inhibition or metabolic therapy, and driving the transformation of this conceptual network into a verifiable, stratifiable, and intervenable framework for cancer treatment.

#### 6.2.2 The Precise Molecular and Cellular Mechanisms Underlying Neuro–Tumor Interactions Remain to be Elucidated

Although a series of original studies have revealed several key mechanisms of neuro‐tumor interactions—including activity‐dependent NLGN3 [214] release and its activation of PI3K–mTOR [215] signaling, AMPA receptor‐ or GABA [99] receptor‐mediated neuron–glioma synapses, the utilization of NMDAR‐related synaptic signaling by brain‐metastasizing cells, and the functional remodeling of host neural circuits by glioblastoma [71, 72, 73, 258, 273]—studies of peripheral solid tumors have also found that the sympathetic and parasympathetic nervous systems regulate tumor initiation, vascular metabolic transition, and dissemination, respectively; cholinergic–NGF positive feedback promotes neural innervation of gastric tumors; loss of tumor suppressor genes [44] can induce neurophhenotypic reprogramming; and Schwann cells [274], circulating neural progenitor cells, and neuroregulated immune cells may also participate in tumor invasion and immune evasion [5, 7, 42, 47, 54].

However, most of these mechanisms stem from specific cancer types, neural subtypes, anatomical locations, and preclinical models, and do not yet constitute a precise molecular and cellular framework with cross‐tumor universality. In particular, it remains unclear how specific patterns of neural activity ultimately determine the proliferation, stemness, migration, and treatment resistance of different malignant cell subpopulations through ligands or neurotransmitters, receptor subtypes, intracellular signaling networks, and metabolic or epigenetic effects. The relative contributions of true synapses, synapse‐like contacts, paracrine signaling, and indirect effects mediated by glial and immune cells at different disease stages also remain to be elucidated. Furthermore, direct causal evidence is still lacking regarding the temporal relationships by which tumor cells reciprocally induce neurogenesis, axonal remodeling, and changes in neural excitability, as well as how these feedback loops form self‐reinforcing circuits at the single‐cell and spatial niche scales.

#### 6.2.3 Multiple Barriers to Clinical Translation

**FIGURE 4:**
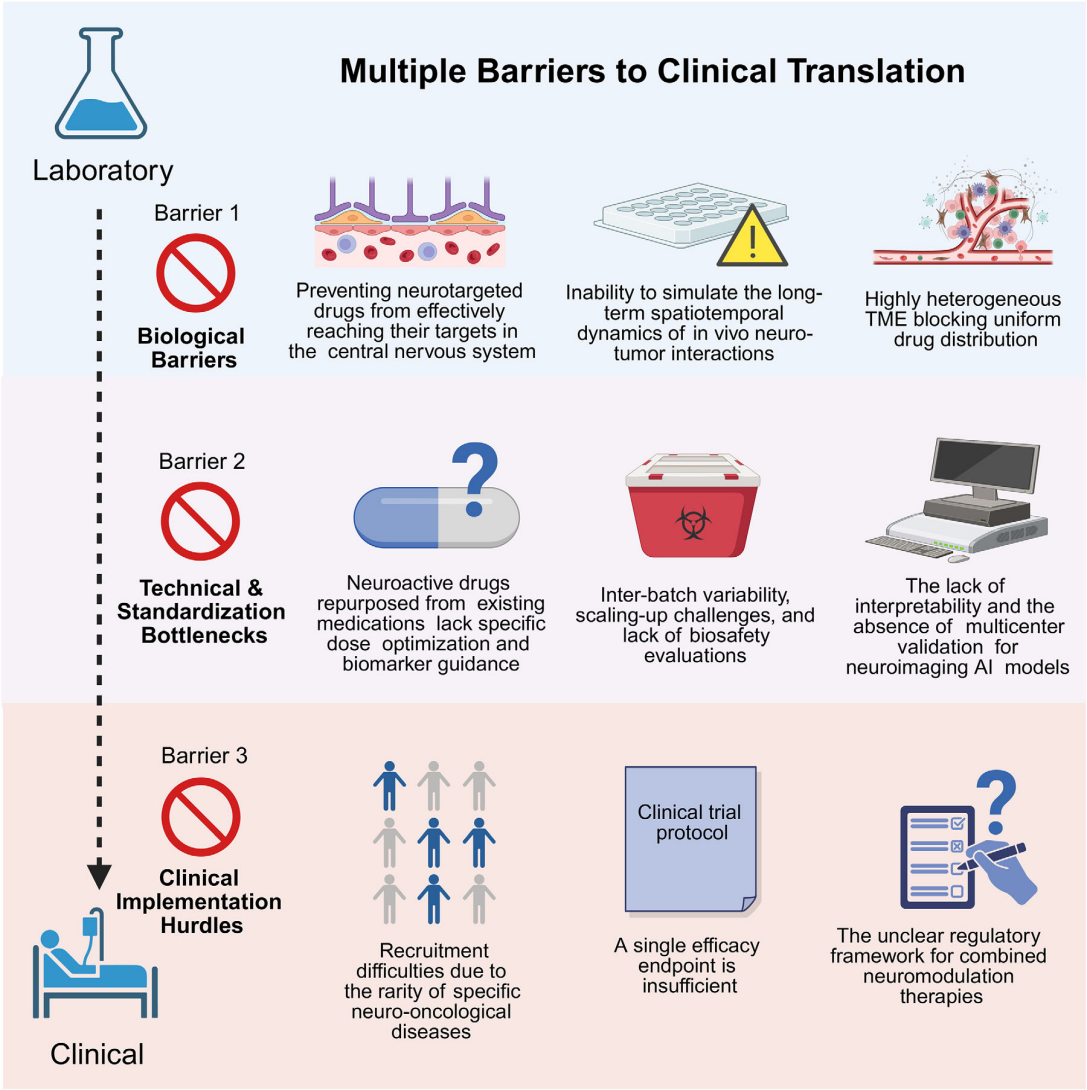
Multiple barriers to clinical translation. **Biological barriers**: The physical barrier function of the blood–brain barrier (BBB) severely limits the efficient delivery of neurotargeted drugs to their targets in the central nervous system. At the same time, traditional 2D in vitro cell culture models are unable to simulate the complex spatiotemporal dynamics of in vivo neuro–tumor interactions over the long term. Furthermore, the highly heterogeneous and dense tumor microenvironment also hinders the uniform distribution of drugs within the tumor. **Technical and standardization bottlenecks**: For neuroactive drugs repurposed from existing medications, there is currently a lack of precise dose optimization protocols and guidance based on specific neurobiological biomarkers. Lipid‐ or polymer‐based nanocarriers face challenges such as significant batch‐to‐batch variability, difficulties in scaling up production, and a lack of biosafety assessments. AI models used for neuroimaging analysis are hampered by their “black box” nature, lacking mechanistic interpretability and extensive validation across multicenter cohorts. **Clinical implementation challenges**: The rarity of certain neuro‐oncology‐related diseases has led to significant difficulties in patient recruitment. Given the complexity of the multidimensional, dynamic interactions within the neuro‐immune–metabolic system, a single clinical trial efficacy endpoint is insufficient to comprehensively evaluate the effectiveness of an intervention. Finally, the clinical regulatory and approval framework for combined neuromodulation therapies remains unclear, delaying the implementation of novel therapies.

Although research on neuro–tumor interactions has achieved breakthroughs in elucidating underlying mechanisms, its translation into clinical practice still faces systemic barriers (Figure 4). Biological barriers pose the primary challenge: the blood–brain barrier, unique to the central nervous system, and the dynamic heterogeneity of the tumor microenvironment severely limit the delivery efficiency of neurotargeted drugs [275]; in vitro models struggle to simulate the spatiotemporal dynamics of in vivo neuro–tumor interactions over the long term, thereby limiting the validation of mechanisms and the assessment of drug efficacy [276].

Significant technical and standardization bottlenecks exist: the repurposing of neuroactive drugs lacks dose optimization and biomarker guidance specific to the neuro‐tumor axis [248, 277]; although nanocarriers hold promise for targeted delivery, inter‐batch variability, challenges in scaling up production, and the absence of biosafety evaluations hinder their clinical advancement [278]; and artificial intelligence models for neuroimaging analysis face challenges related to a lack of interpretability and the absence of multicenter validation [279]. Challenges in clinical implementation are particularly pronounced: the rarity of neuro‐oncological diseases makes patient recruitment difficult; traditional clinical trial designs struggle to capture the dynamic effects of neuromodulation on the immune microenvironment; and the complexity of multidimensional neuro‐immune‐metabolic interactions means that a single endpoint cannot fully reflect the efficacy of neuromodulatory interventions [280]; furthermore, the regulatory framework has not yet established a clear approval pathway for combined neuromodulation therapies [278, 279]. In addition, the lack of interdisciplinary collaboration mechanisms and insufficient clinical validation of neuropathological features as stratification markers further delay the translational process [281].

### 6.3 Future Directions

Future progress in cancer neuroscience will require more physiologically relevant experimental systems to define the complex tumor–neural landscape and evaluate emerging therapeutic targets. Traditional two‐dimensional culture systems cannot adequately reproduce the spatial and temporal dynamics of neuro–immune–tumor interactions. Modern neurobiological approaches should therefore be increasingly integrated into oncology research. These approaches include intact‐tissue spatial transcriptomics for mapping the perineural niche [47, 272], patient‐derived organoid co‐cultures containing functional neuronal networks, and electrophysiological patch‐clamp recordings [273, 274].

The combined application of these methods may help determine whether tumor cells are functionally integrated into neural circuits rather than merely located near neuronal structures. This distinction is important because spatial proximity alone does not establish functional neural–tumor communication. Future studies should therefore focus on identifying direct functional interactions between tumor cells and neural networks and on clarifying how these interactions contribute to tumor progression and treatment resistance.

Clinical translation will also require prospective validation of candidate neuro‐oncological interventions. Although retrospective evidence suggests that β‐blocker use may enhance responses to immunotherapy [209], this association remains to be confirmed in prospective, biomarker‐based clinical trials. A major priority is therefore the identification of noninvasive biomarkers that can be used to select patients who are most likely to benefit from neuro‐oncological interventions.

Potential stratification tools include circulating neurotrophic factors, spatial multi‐omics signatures, and advanced neuroimaging techniques, including resting‐state functional magnetic resonance imaging and PET‐CT‐based assessment of synaptic density [123]. These approaches may help identify tumors that are particularly dependent on neural signaling and may support the development of more individualized therapeutic strategies.

In the long term, determining how tumors acquire functional roles within neural networks may reveal system‐level vulnerabilities that contribute to cancer resistance. Identifying these vulnerabilities may ultimately support the development of more durable and sustainable treatment approaches [2].

## Author Contributions

**Mingxuan Xie**: conceptualization, investigation, writing – original draft, visualization. **Yu Zhao**: conceptualization, investigation, writing – original draft. **Xinruo Xing**: investigation, writing – original draft, visualization. **Yuqiang Liu**: writing – review and editing, validation. **Jungang Zhao**: supervision, writing – review and editing. **Maojin Yao**: supervision, writing – review and editing. **Jun Tang**: funding acquisition, supervision, writing – review and editing. **Jianzhu Zhao**: conceptualization, funding acquisition, supervision, project administration, writing – review and editing. Mingxuan Xie, Yu Zhao, and Xinruo Xing contributed equally. All authors have read and approved the final manuscript. All authors have read and approved the final manuscript.

## Funding

This review is supported by General Project of Natural Science Foundation of Liaoning Provincial Department of Science and Technology (No. 2025‐MS‐233); General Project of Natural Science Foundation of Liaoning Provincial Department of Science and Technology (No. 2022‐MS‐09); General Project of Natural Science Foundation of Xizang Provincial Department of Science and Technology (No. XZ2021R‐ZY46(Z)); the Guangzhou Science and Technology Plan Project‐2024 Municipal School (Institute) Enterprise Joint Funding Project (2024A03J1221); the Tibet Natural Science Foundation of China (XZ2021ZR‐ZY45(Z)).

## Ethics Statement

The authors have nothing to report.

## Conflicts of Interest

The authors declare no conflicts of interest.

## Data Availability

The authors have nothing to report.
